# Synthesis and Characterization of 2-(((2,7-Dihydroxynaphthalen-1-yl)methylene)amino)-3′,6′-bis(ethylamino)-2′,7′-dimethylspiro[isoindoline-1,9′-xanthen]-3-one and Colorimetric Detection of Uranium in Water

**DOI:** 10.3390/m1725

**Published:** 2023-09-15

**Authors:** Rahisa Mohammed, Peace Ogadi, Dennis M. Seth, Amrutaa Vibho, Sarah K. Gallant, Rory Waterman

**Affiliations:** 1 Department of Chemistry and Biochemistry, Norwich University, Northfield, VT 05663, USA; 2 Department of Chemistry, University of Vermont, Burlington, VT 05405, USA

**Keywords:** Schiff base, ligand, colorimetric analysis

## Abstract

2-(((2,7-Dihydroxynaphthalen-1-yl)methylene)amino)-3′,6′-bis(ethylamino)-2′,7′-dimethyl-spiro[isoindoline-1,9′-xanthen]-3-one was synthesized using Rhodamine 6G hydrazide (prepared using literature methods) and commercially available 2,7-dihydroxynaphthalene-1-carbaldehyde via imine condensation. Structural characterization was performed using FT-IR, ^1^H-NMR, ^13^C-NMR, X-ray, and HRMS. This Schiff base shows promise as a ligand for the colorimetric analysis of uranium in water.

## 1. Introduction

Schiff bases are a class of compounds derived from the reaction of primary amines with carbonyl compounds, resulting in the formation of an imine (C=N) [1]. Structurally, these compounds are well suited for binding transition metals [2–4], and Schiff bases have commonly been used as fluorescent or colorimetric probes for metal ions [5,6]. Due to their modular synthesis, these molecules are highly tunable, and varying the structure has been shown to result in high selectivity for a particular metal ion in solution, even in the presence of competing metal ions [6].

Industrially, Schiff bases have been used as catalysts, dyes, or stabilizers [7]. These compounds have also been thoroughly investigated for their high levels of biological activity as metal complexes [8–10] or as small-molecule candidates for anticancer activity [11], as well as for antiviral, antibacterial, and anti-inflammatory properties, among others [7].

The following work demonstrates the synthesis and characterization of 2-(((2,7-dihydroxynaphthalen-1-yl)methylene)amino)-3′,6′-bis(ethylamino)-2′,7′-dimethylspiro[isoindoline-1,9′-xanthen]-3-one (**PROM1**), a Schiff base synthesized from Rhodamine 6G hydrazide and 2,7-dihydroxynaphthalene-1-carbaldehyde. A related Schiff base was previously reported and shown to have high uranium binding affinity [12]. **PROM1** was also tested as a ligand for the colorimetric sensing of uranium in water.

## 2. Results and Discussion

### 2.1. Synthesis of 2-(((2,7-Dihydroxynaphthalen-1-yl)methylene)amino)-3′,6′-bis(ethylamino)-2′,7′-dimethylspiro[isoindoline-1,9′-xanthen]-3-one (PROM1)

**Scheme 1.**
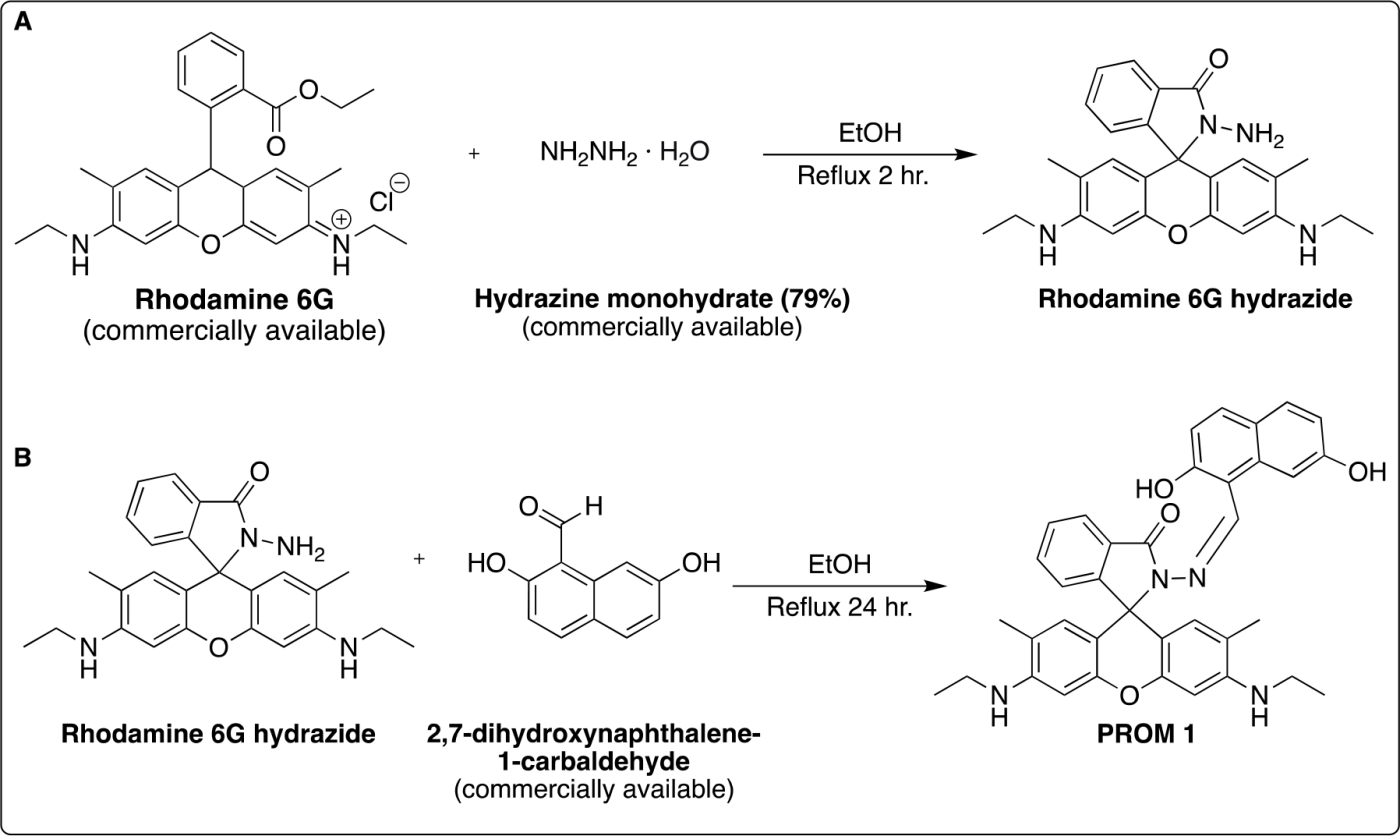
(**A**) Synthesis of Rhodamine 6G hydrazide via literature procedure [13]. (**B**) Synthesis of **PROM1** ligand.

Schiff base **PROM1** was synthesized in two steps. First, Rhodamine 6G hydrazide was prepared using literature methods (Scheme 1A) [13]. This starting material was then used for imine condensation with commercially available 2,7-dihydroxynaphthalene-1-carbaldehyde via overnight reflux (Scheme 1B).

**Figure 1.**
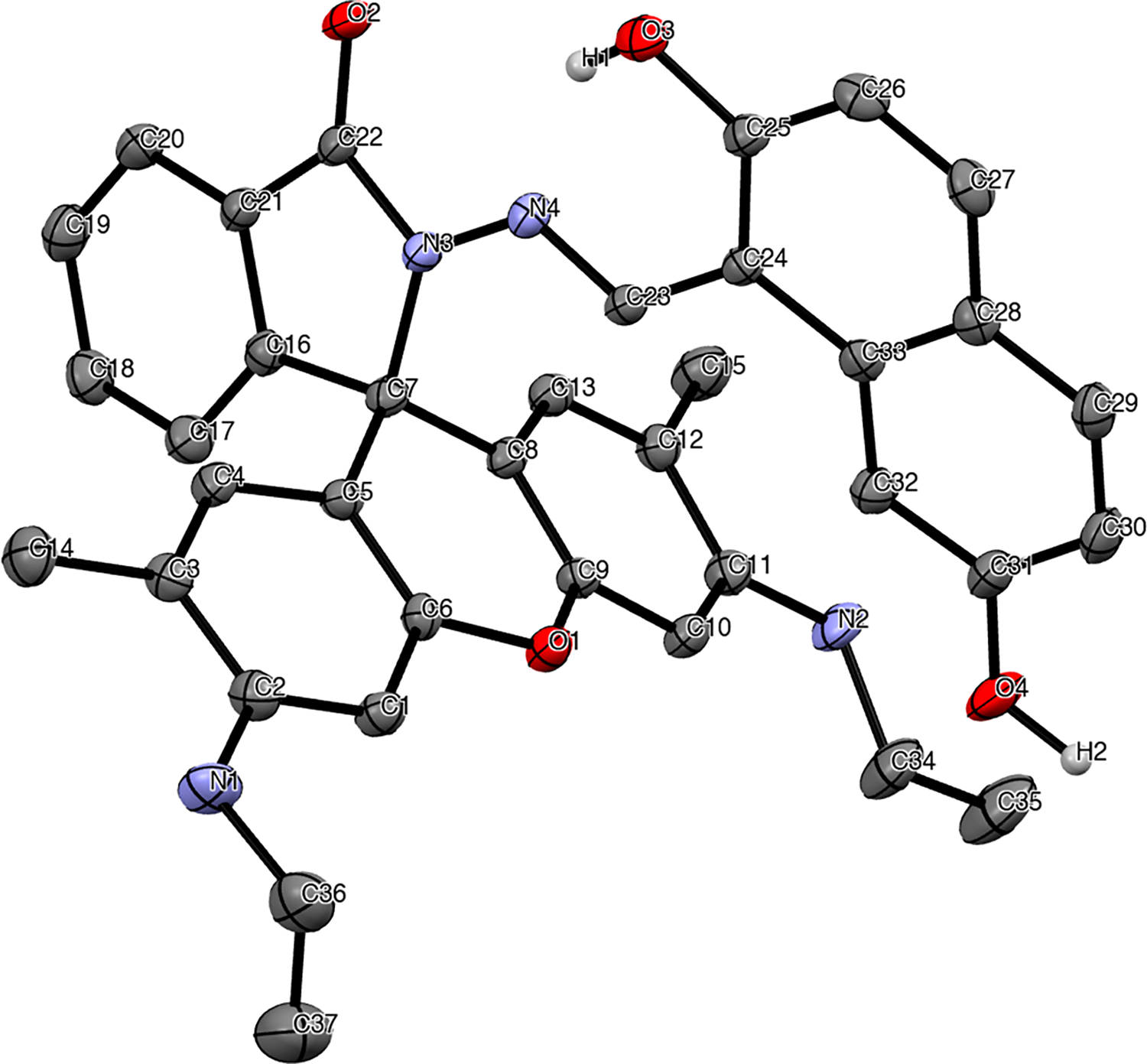
Molecular structure of **PROM1**•2 MeCN with thermal ellipsoids drawn at a 30% probability level. The solvent molecules are not shown, and H atoms are omitted, except for on heteroatoms for clarity.

**PROM1** was recrystallized via slow evaporation in a mixture of acetonitrile and ethanol. The crystalline product was analyzed using X-ray, FT-IR, high-resolution ESI-MS, and ^1^H- and ^13^C-NMR (for spectra, see Supplementary Materials, Figures S1–S6). The structural determination of **PROM1** was performed via X-ray crystallography (Figure 1), and the structure was confirmed via NMR spectroscopy.

### 2.2. Colorimetric Detection of Uranium

Uranium is a chemically and radiologically toxic element that occurs naturally in groundwater, and may have increased concentrations due to mining operations, processing for nuclear power, or improper nuclear waste management [14]. A simple colorimetric test for the presence of uranium in drinking water may prevent accidental exposure to uranium, which can result in kidney disease, kidney failure, or cancer [15].

**Figure 2.**
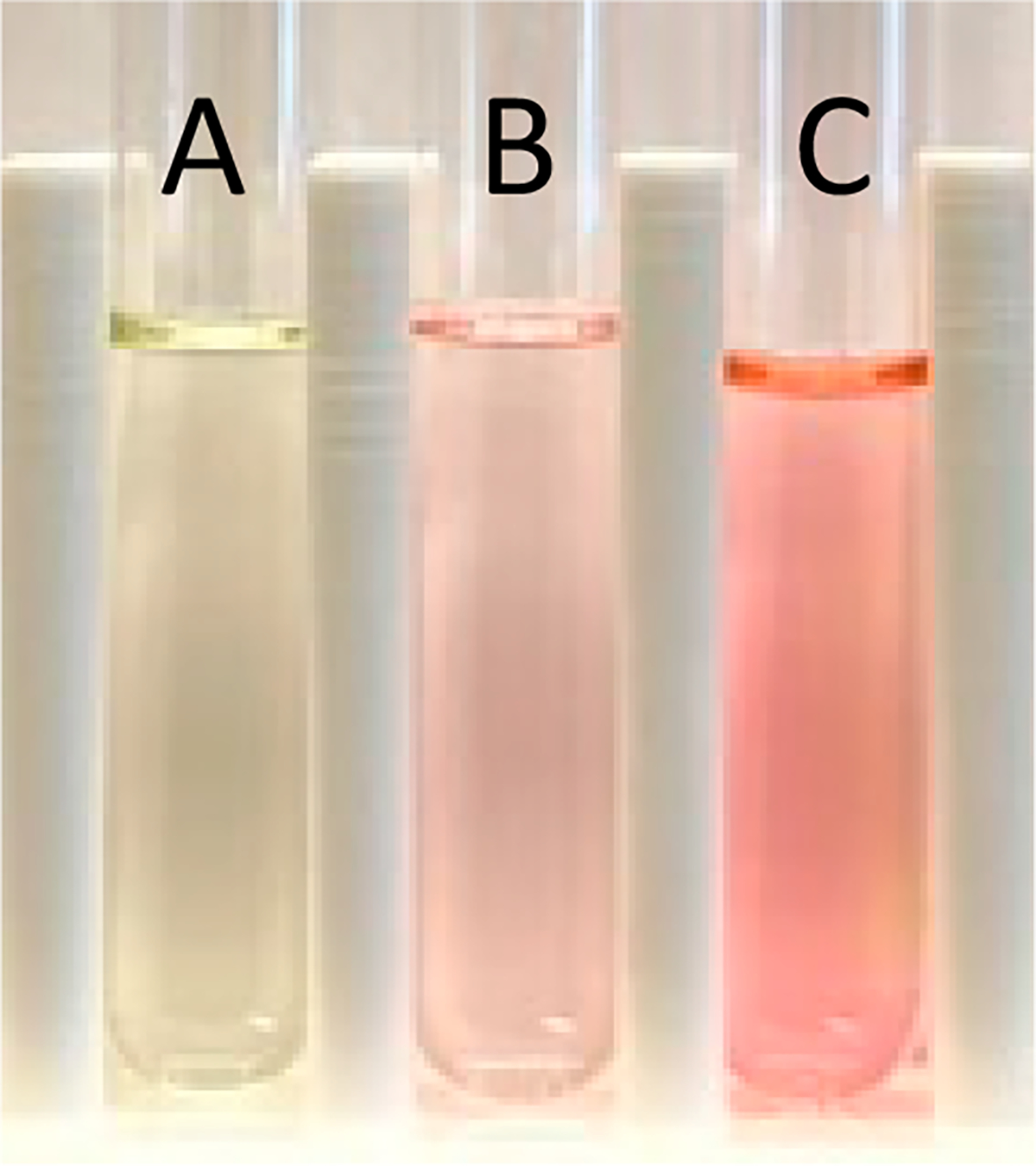
(**A**) A stock solution of **PROM1** in DMSO. (**B**) **PROM1** and 500 μg/L uranyl nitrate in 50/50 DMSO and water. (**C**) **PROM1** and 5000 μg/L uranyl nitrate in 50/50 DMSO and water.

A stock solution of **PROM1** was prepared in dimethylsulfoxide (DMSO). When mixed with aqueous solutions of uranyl nitrate, a color change from yellow to pink was observed (Figure 2).

## 3. Materials and Methods

### 3.1. General

All starting materials were purchased from commercial suppliers. Rhodamine 6G and 2,7-dihydroxynaphthalene-1-carbaldehyde were purchased from Sigma Aldrich (Burlington, MA, USA), 80% hydrazine from TCI (VWR), and uranyl nitrate hexahydrate from Fisher Scientific (Pittsburgh, PA, USA). Solvents were reagent/ ACS-grade and were purchased from VWR, Radnor, PA, USA. Rhodamine 6G hydrazide was synthesized as described previously in the literature [13].

The IR spectrum was recorded on a Perkin Elmer Spectrum 100 FT-IR, UV-Vis spectra were recorded on a Shimadzu UV-2600i, mass spectrometry was run via direct infusion in pos ESI on an Agilent 6530 QToF HRMS, NMR spectra were collected on a Bruker AXR 500 MHz spectrometer in dimethylsulfoxide-*d*_6_ solution with reference to residual solvent signals (DMSO-*d*_6_, δ = 2.50 ppm for ^1^H and 39.52 ppm for ^13^C). X-ray diffraction data were collected on a Bruker APEX 2 CCD platform diffractometer [Mo Kα (λ = 0.71073 Å)].

### 3.2. 2-(((2,7-Dihydroxynaphthalen-1-yl)methylene)amino)-3′,6′-bis(ethylamino)-2′,7′-dimethylspiro[isoindoline-1,9′-xanthen]-3-one (PROM1)

To Rhodamine 6G hydrazide (0.1915 g, 0.45 mmol), ethanol (15 mL) and acetic acid (5–6 drops) were added. The mixture was stirred to dissolve, and then 2,7-dihydroxynaphthalene-1-carbaldehyde (0.0837 g, 0.44 mmol) was added. The resulting solution was stirred for 24 h under reflux at 72 °C. The yellow solid product was separated via hot vacuum filtration (0.1839 g, 68.8%). A portion of the product was redissolved in warm acetonitrile, layered with ethanol, and recrystallized via slow evaporation at 5 °C. The yellow crystalline product was used directly for X-ray, ^1^H and ^13^C NMR, and HRMS. HRMS *m*/*z* calcd. for C_37_H_34_N_4_O_4_: 598.26. Found [M + H]^+^: 599.2641. ^1^H-NMR (DMSO-*d*_6_): 9.57 (s, 1H, imine-H), 8.00 (d, *J* = 7.3, 1H, Ar-H), 7.73 (d, *J* = 8.9, 1H, Ar-H), 7.70–7.63 (m, 3H, Ar-H), 7.13–7.11 (overlapping m, 2H, Ar-H), 6.95 (dd, *J* = 8.70, 1.96, 1H, Ar-H), 6.82 (d, *J* = 8.9, 1H, Ar-H), 6.40 (s, 2H, Ar-H), 6.27 (s, 2H, Ar-H), 3.46 (residual water), 3.16 (q, *J* = 6.58, 4H, N-**CH**_**2**_CH_3_), 2.54 (DMSO), 2.10 (s, MeCN), 1.87 (s, 6H, Ar-CH_3_), 1.22 (t, *J* = 7.09, 6H N-CH_2_**CH**_**3**_), 1.09 (t, *J* = 7, EtOH). ^13^C-NMR (DMSO-*d*_6_): 163.5 (quat C, carbonyl), 158.5 (quat Ar), 157.4 (quat Ar), 151.4 (quat Ar), 151.2 (quat Ar), 148.1 (quat Ar), 147.2 (imine CH), 134.1 (Ar CH), 133.7 (quat Ar), 133.1 (Ar CH), 130.8 (Ar CH), 129.0 (quat Ar), 128.6 (Ar CH), 127.0 (Ar CH), 124.0 (Ar CH), 123.1 (Ar CH), 122.2 (quat Ar), 118.7 (quat Ar), 118.1 (CH_3_**CN**), 115.6 (Ar CH), 115.2 (Ar CH), 107.0 (quat Ar), 103.9 (quat Ar), 102.7 (Ar CH), 95.7 (Ar CH), 65.6 (quat C), 56.1 (EtOH), 39.5 (q, DMSO), 37.5 (CH_2_), 18.6 (EtOH), 17.0 (CH_3_), 14.1 (CH_3_), 1.22 (**CH**_**3**_CN). FT-IR (ν, cm^−1^): 3440.96 (ν_NH_), 3300.00 (ν_OH_), 2964.17 (ν_CH_), 2866.53 (ν_CH_), 1694.78 (ν_CO_), 1617.61 (ν_CH_), 1564.92, 1515.18, 1465.34, 1449.02, 1420.64, 1379.85, 1348.29, 1321.63, 1270.01, 1217.86, 1198.74, 1159.27, 1138.86, 1086.14, 1035.71, 1013.46, 964.98, 923.35, 896.72, 876.22, 859.07, 841.39, 830.84, 811.81, 791.70.

### 3.3. X-ray Data

A suitable yellow crystal, grown from acetonitrile and ethanol, was mounted on a MiTiGen micromount with Paratone-N cryoprotectant oil. The structure was solved via direct methods using SHELXT and refined via full-matrix least-squares methods against *F*^2^ by SHELXL-2018/3 [16,17]. All non-hydrogen atoms were refined with anisotropic displacement parameters. All hydrogen atoms were refined with isotropic displacement parameters. H1 and H2 were refined freely. Hydrogen atoms on carbon were included in calculated positions and were refined using a riding model.

Crystallographic data for the structure reported here have been deposited with the Cambridge Crystallographic Data Centre [18]. CCDC 2287758 contain the supplementary crystallographic data for this manuscript. These data can be obtained free of charge from The Cambridge Crystallographic Data Centre via www.ccdc.cam.ac.uk/structures. The final CIF file was generated using FinalCif [19].

Crystal data for C_41_H_40_N_6_O_4_ (M = 680.79 g/mol): triclinic, space group P–1 (no. 2), a = 11.37(3) Å, b = 11.51(3) Å, c = 14.38(3) Å, α = 80.06(3)°, β = 74.82(3)°, γ = 85.75(3)°, V = 1789(7) Å^3^, Z = 2, T = 150(2) K, μ(MoKα) = 0.083 mm^−1^, D_calc_ = 1.264 g/cm^3^, 14,714 reflections measured (3.59° ≤ 2Θ ≤ 47.48°), 5320 unique (Rint = 0. 0799, Rsigma = 0.0952), which were used in all calculations. The final R1 was 0.0450 (I > 2σ(I)), and wR2 was 0.1066 (all data).

### 3.4. Colorimetric Analysis of Uranium Binding

Stock solutions of uranyl nitrate in water were prepared by dissolving the appropriate amounts of solid uranyl nitrate hexahydrate in deionized water. The **PROM1** stock solution was prepared by dissolving 0.0200 g (0.033 mmol) in 100 mL of DMSO. Equal amounts of the ligand stock solution (in DMSO) and uranyl nitrate hexahydrate solution (in water) were then mixed resulting in the yellow to pink color change upon uranium binding (Figure 2).

## 4. Conclusions

Novel Schiff base 2-(((2,7-dihydroxynaphthalen-1-yl)methylene)amino)-3′,6′-bis(ethylamino)-2′,7′-dimethylspiro[isoindoline-1,9′-xanthen]-3-one (**PROM1**) was synthesized from Rhodamine 6G hydrazide and 2,7-dihydroxynaphthalene-1-carbaldehyde. **PROM1** was shown to bind uranium in water and is a plausible ligand for the colorimetric analysis of uranium in drinking water.

## Supplementary Material

Check Cif

Supplemental Materials

CIF

Mol Document

3D Structure Mol

## Data Availability

The authors confirm that the data supporting the findings of this study are available within the article and/or its Supplemental Materials.
