# Supplementary material for: Synthesis and Characterization of 2-(((2,7-Dihydroxynaphthalen-1-yl)methylene)amino)-3′,6′-bis(ethylamino)-2′,7′-dimethylspiro[isoindoline-1,9′-xanthen]-3-one and Colorimetric Detection of Uranium in Water
Source: Molbank. Author manuscript; Available in PMC 2023 Sep 29. (PMC10540563; doi:10.3390/m1725)
Supplement: Supplemental Materials [file NIHMS1933183-supplement-Supplemental_Materials.pdf]

molbank

Supporting Information

Synthesis and Characterization of 2-(((2,7-Dihydroxynaphthalen-1-yl)methylene)amino)-3′,6′-bis(ethylamino)-2′,7′-dimethylspiro[isoindoline-1,9′-xanthen]-3-one and Colorimetric Detection of Uranium in Water

Rahisa Mohammed [1], Peace Ogadi [1], Dennis M. Seth Jr. [2], Amrutaa Vibho [1], Sarah K. Gallant [1,*] and Rory Waterman [2]

[1] Department of Chemistry and Biochemistry, Norwich University, VT, USA
[2] Department of Chemistry, University of Vermont, VT, USA; rory.waterman@uvm.edu (R.W.)
* Correspondence: sgallant@norwich.edu

This file contains:

Nuclear Magnetic Resonance Spectra 2

IR Spectrum 4

Mass Spectrum 4

Color Change Due to pH vs Uranium Binding 5

NMR Spectra

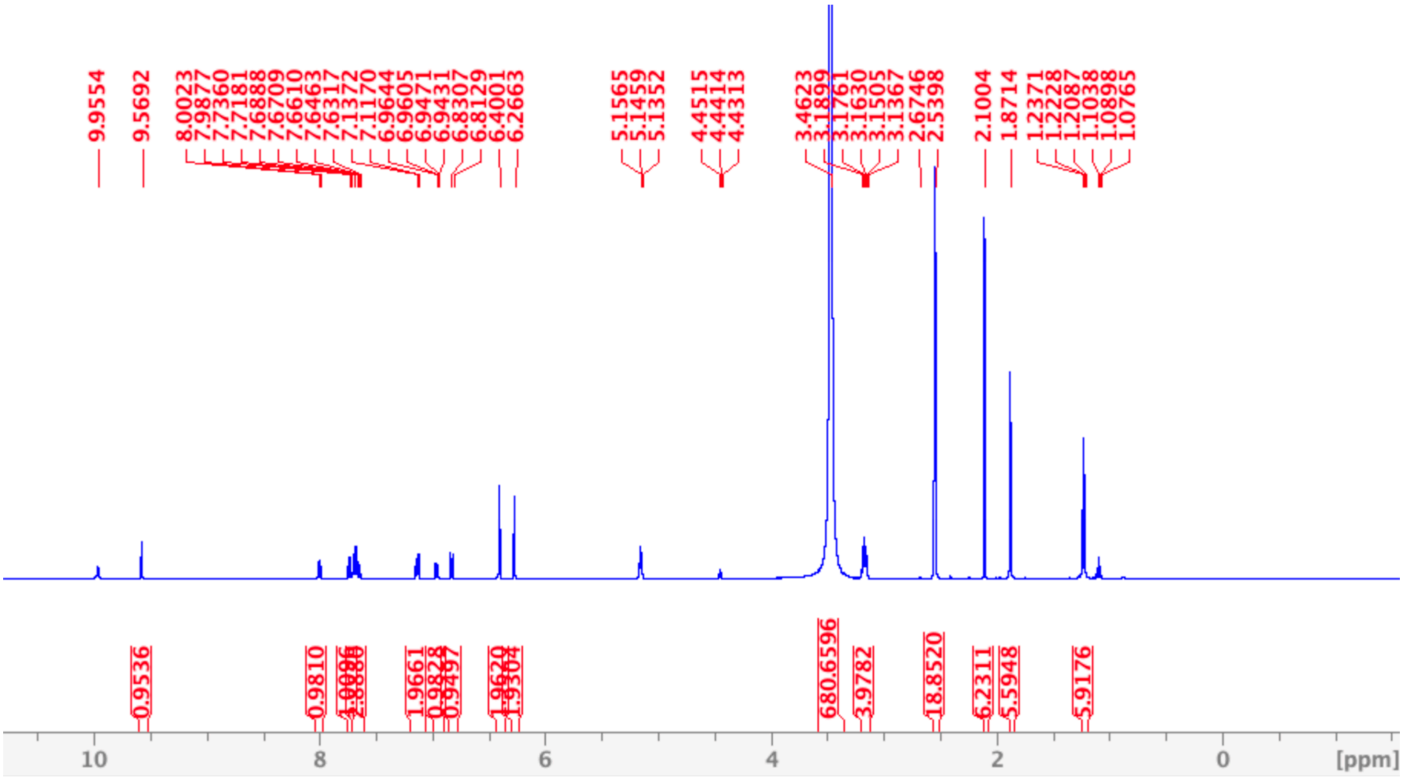

Figure S1. ^{1}H-NMR spectrum of **PROM1** (600 MHz, DMSO-d_6).

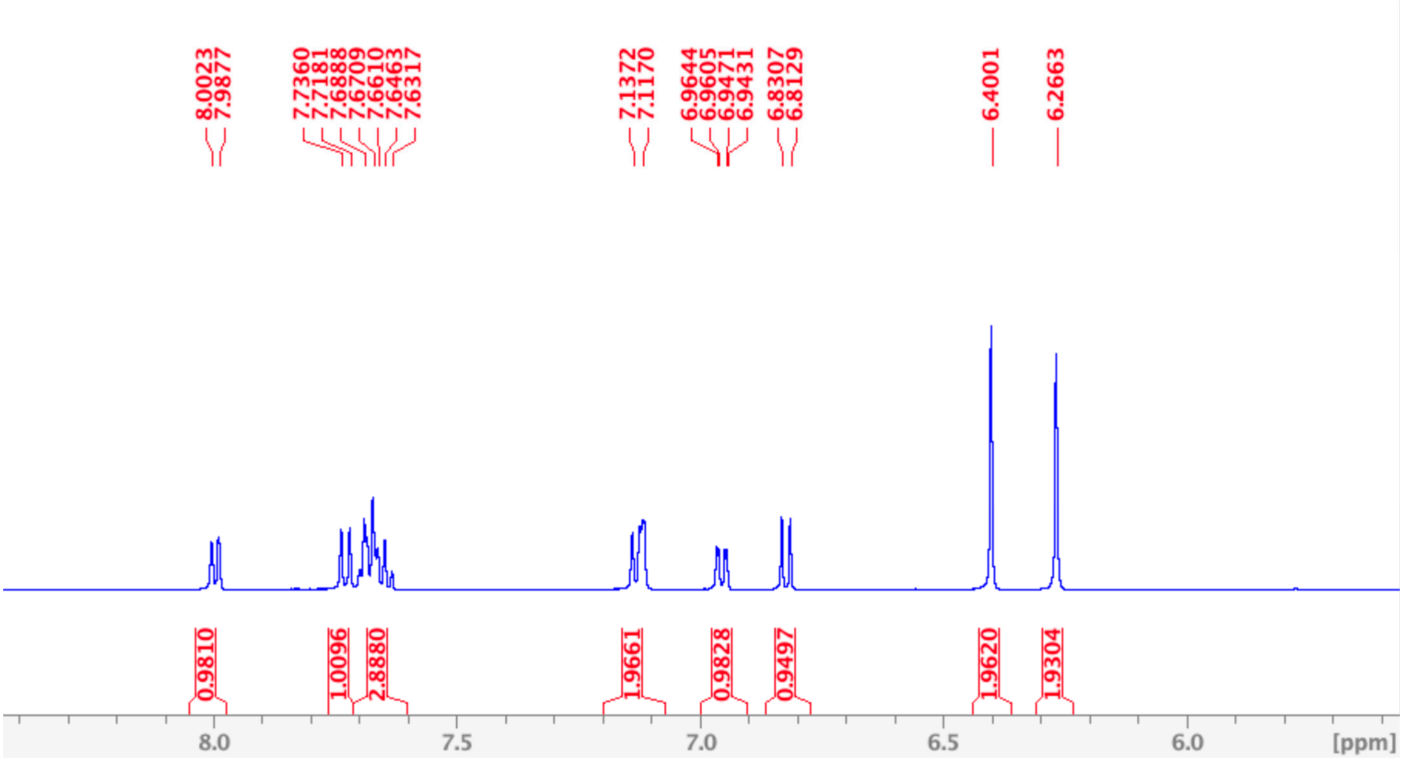

Figure S2. An inset of the ^{1}H-NMR spectrum of **PROM1** from 6.0 to 8.5 ppm (600 MHz, DMSO-d_6).

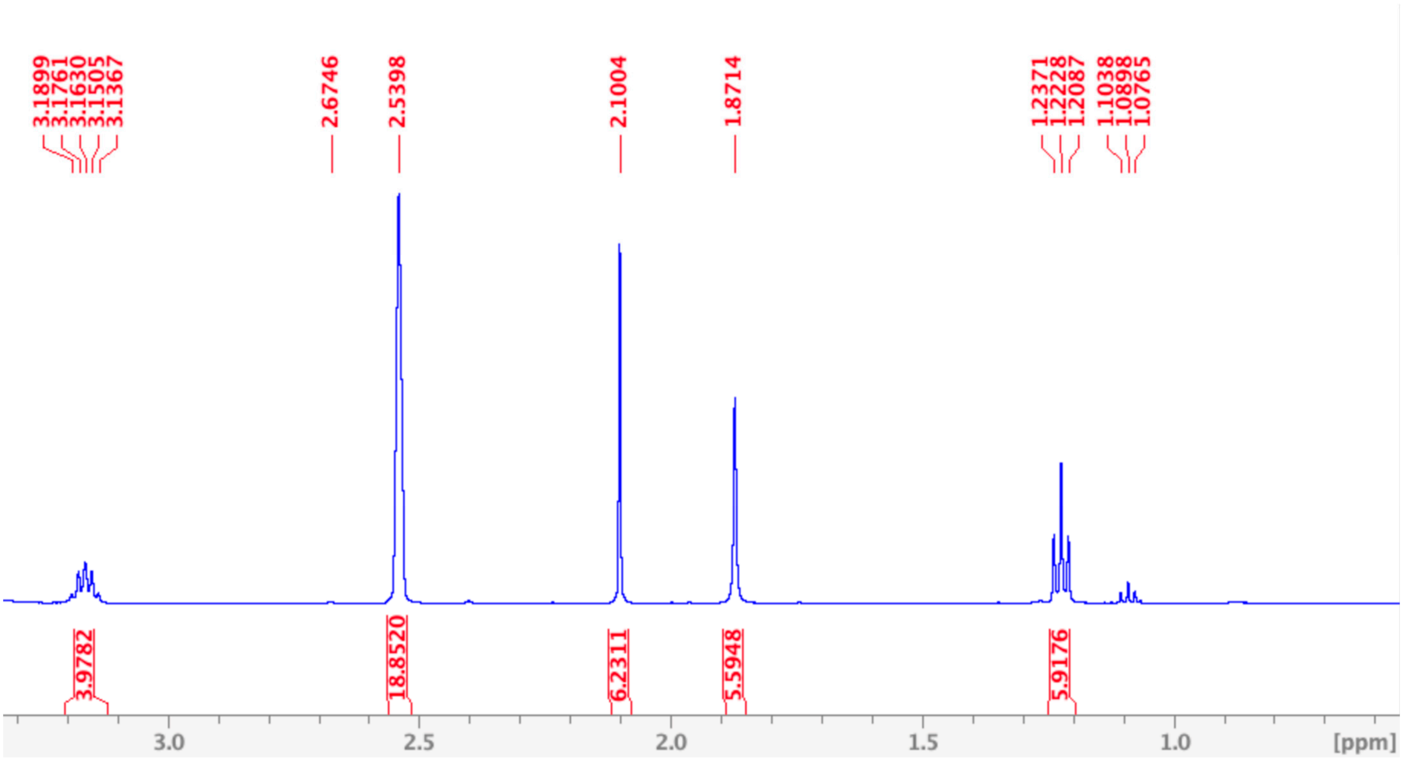

Figure S3. An inset of the ^{1}H-NMR spectrum of **PROM1** from 1.0 to 4.0 ppm (600 MHz, DMSO-d$_6$).

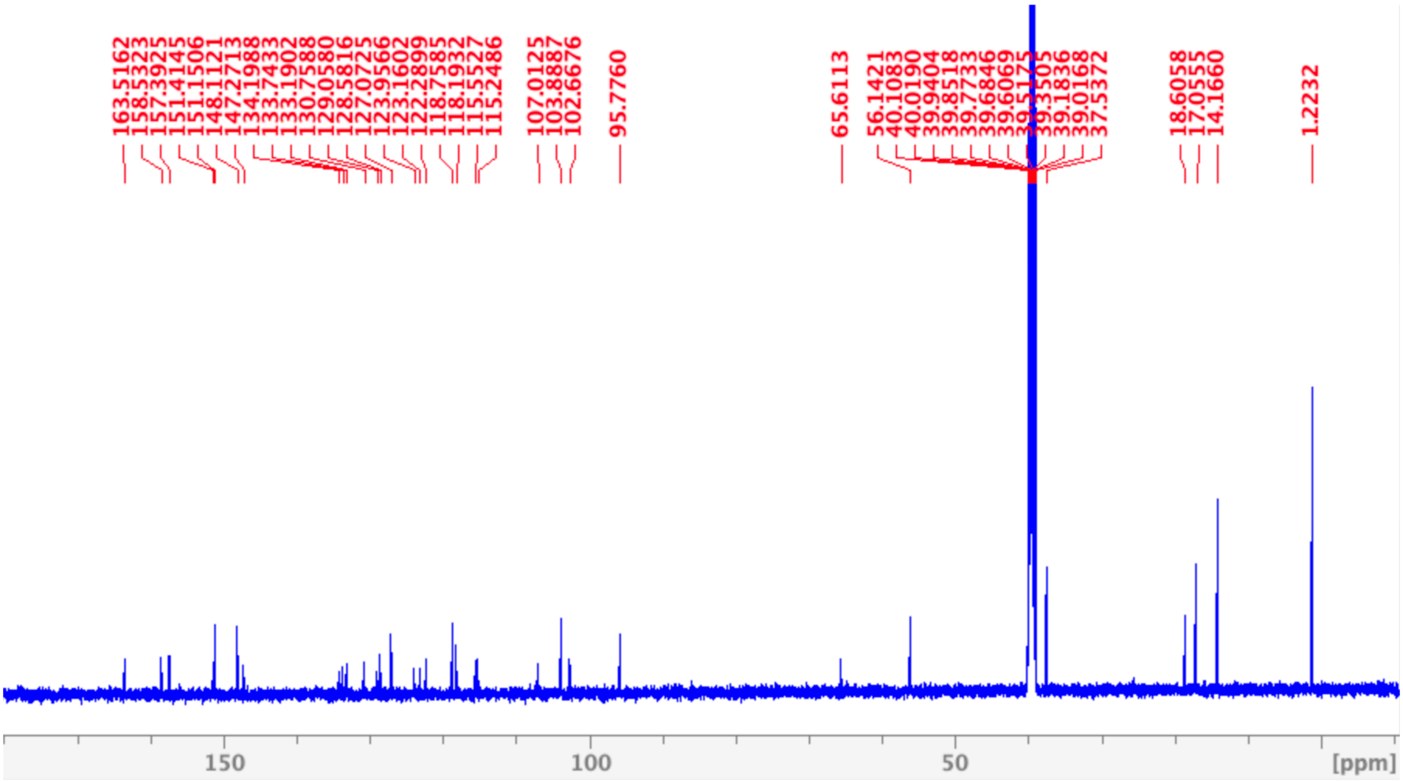

Figure S4. ^{13}C-NMR spectrum of **PROM1** (600 MHz, DMSO-d$_6$).

IR Spectrum

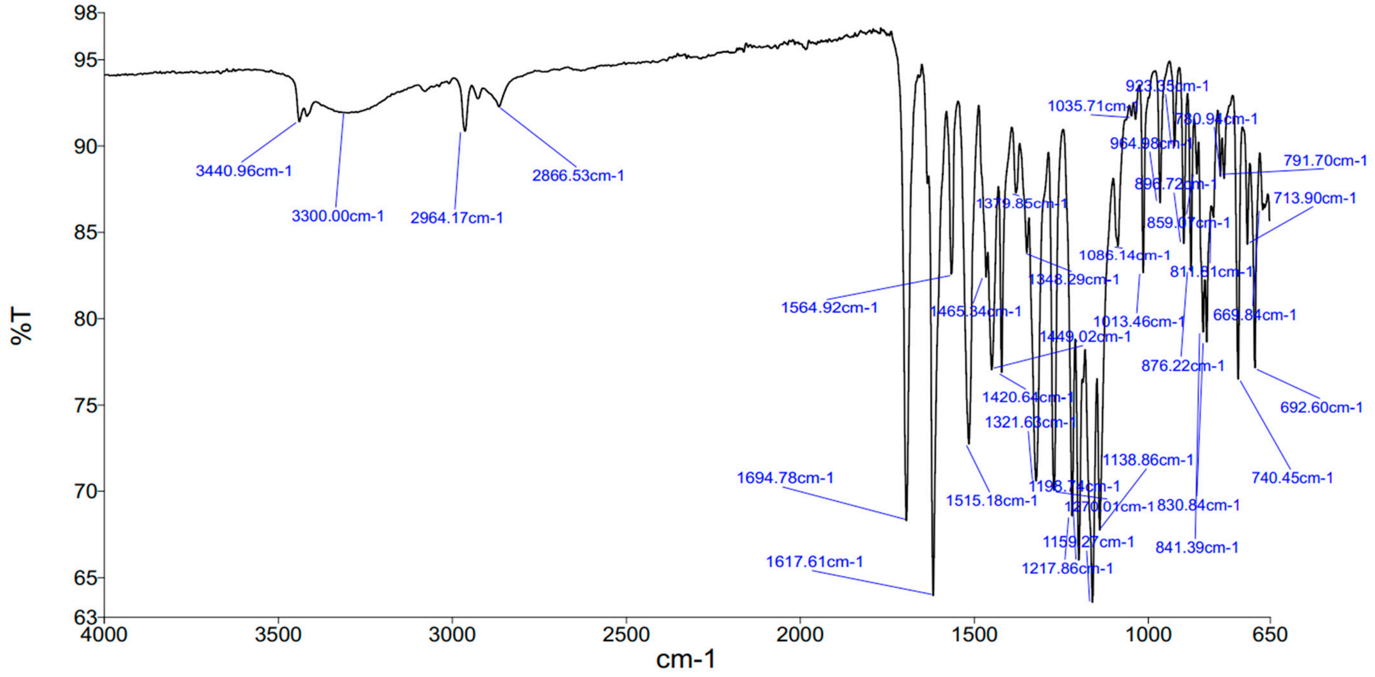

Figure S5. IR spectrum of **PROM1**.

Mass Spectrum

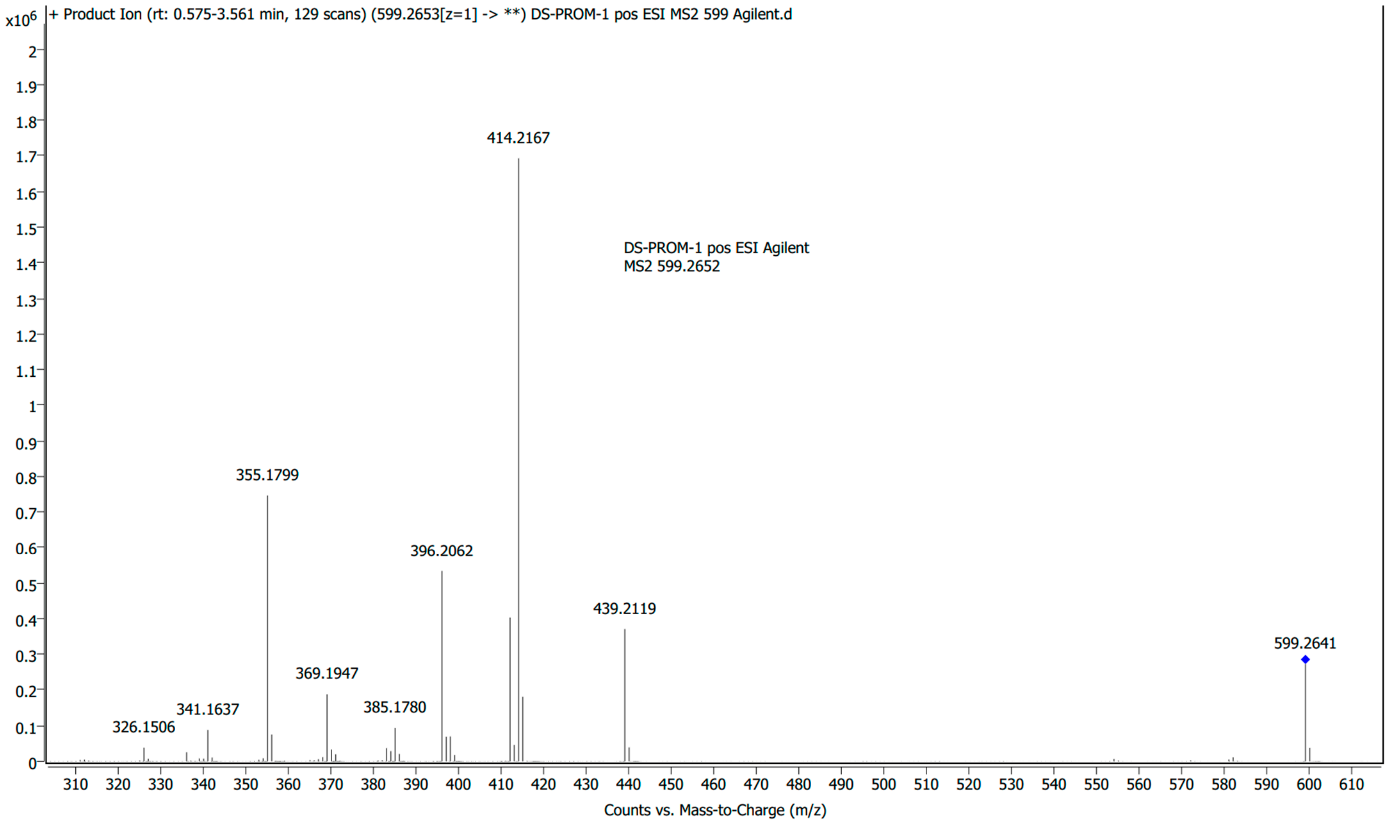

Figure S6. High-resolution mass spectrum of **PROM1**. Observed protonated molecular ion (MH^+).

Color Change Due to pH vs Uranium Binding

An experiment was conducted to demonstrate the difference in color change upon acidification of the ligand in comparison to the color change upon uranium binding. **PROM1** and uranium stock solutions were each determined to have a pH of 6, before and after mixing. Three test tubes of **PROM1** in DMSO/water were prepared to test for color change due to ligand acidification. Test tube A was a mixture of PROM1 in DMSO/water. To tube B, which originally contained a mixture of DMSO/water, 0.3 M nitric acid was added dropwise until color change was observed (final pH 3). Tube C was **PROM1** and 5,000 µg/L uranyl nitrate in 50/50 DMSO and water, which has a pH of 6 after mixing. This experiment demonstrates that the color change observed at pH 6 is due to uranium binding, while intense color change observed at pH 3 is due to the opening of the ligand spirolactam ring by protonation.

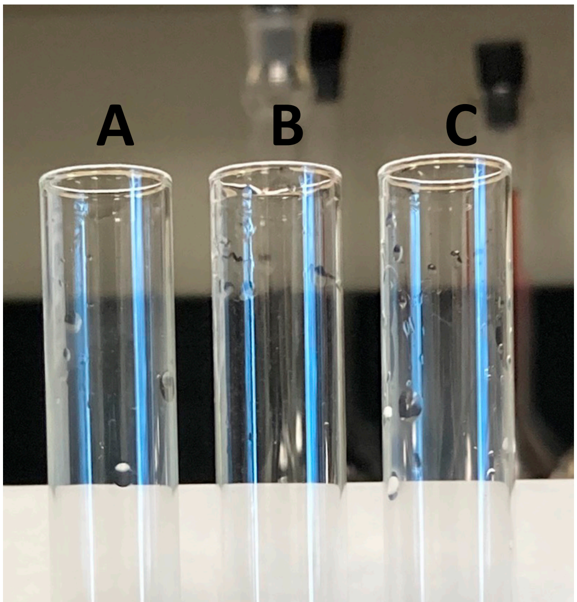

Figure S7. A) A stock solution of **PROM1** in DMSO/water with pH of 6. B) A stock solution of **PROM1** adjusted to pH 3 with 0.3 M HNO_3. C) **PROM1** and 5,000 µg/L uranyl nitrate in 50/50 DMSO and water.
