# Supplementary material for: Synthesis and Characterization of 2-(((2,7-Dihydroxynaphthalen-1-yl)methylene)amino)-3′,6′-bis(ethylamino)-2′,7′-dimethylspiro[isoindoline-1,9′-xanthen]-3-one and Colorimetric Detection of Uranium in Water
Source: Molbank. Author manuscript; Available in PMC 2023 Sep 29. (PMC10540563; doi:10.3390/m1725)

checkCIF/PLATON report

Structure factors have been supplied for datablock(s) DS_4_203c_0m_a

THIS REPORT IS FOR GUIDANCE ONLY. IF USED AS PART OF A REVIEW PROCEDURE FOR PUBLICATION, IT SHOULD NOT REPLACE THE EXPERTISE OF AN EXPERIENCED CRYSTALLOGRAPHIC REFEREE.

No syntax errors found. CIF dictionary Interpreting this report

Datablock: DS_4_203c_0m_a

```
Bond precision:   C-C = 0.0054 A          Wavelength=0.71073

Cell:            a=11.37(3)         b=11.51(3)        c=14.38(3)
                 alpha=80.06(3)     beta=74.82(3)     gamma=85.75(3)
Temperature:     150 K

                Calculated                      Reported
Volume          1788(8)                         1789(7)
Space group     P -1                            P -1
Hall group      -P 1                            -P 1
Moiety formula  C37 H34 N4 O4, 2(C2 H3 N)      C41H40N6O4
Sum formula     C41 H40 N6 O4                   C41 H40 N6 O4
Mr              680.79                          680.79
Dx,g cm-3       1.265                           1.264
Z               2                               2
Mu (mm-1)       0.083                           0.083
F000            720.0                           720.0
F000'           720.29
h,k,lmax        12,13,16                        12,12,16
Nref            5419                            5320
Tmin,Tmax       0.988,0.994                     0.662,0.745
Tmin'           0.986

Correction method= # Reported T Limits: Tmin=0.662 Tmax=0.745
AbsCorr = MULTI-SCAN

Data completeness= 0.982            Theta(max)= 23.742

R(reflections)= 0.0450( 2794)                   wR2(reflections)=
                                                0.1066( 5320)
S = 0.897                  Npar= 479
```

The following ALERTS were generated. Each ALERT has the format
test-name_ALERT_alert-type_alert-level.
Click on the hyperlinks for more details of the test.

Alert level B

```
THETM01_ALERT_3_B  The value of sine(theta_max)/wavelength is less than 0.575
            Calculated sin(theta_max)/wavelength =    0.5665
```

Author Response: The run was done with 60 second frames; the crystal was small and weakly diffracting.

Alert level C

```
PLAT042_ALERT_1_C Calc. and Reported MoietyFormula Strings  Differ     Please Check
PLAT148_ALERT_3_C s.u. on the      a    - Axis is (Too) Large ....      0.030 Ang.
PLAT148_ALERT_3_C s.u. on the      b    - Axis is (Too) Large ....      0.030 Ang.
PLAT148_ALERT_3_C s.u. on the      c    - Axis is (Too) Large ....      0.030 Ang.
PLAT220_ALERT_2_C NonSolvent   Resd 1  C   Ueq(max)/Ueq(min) Range        3.6 Ratio
PLAT222_ALERT_3_C NonSolvent Resd 1  H   Uiso(max)/Uiso(min) Range        4.1 Ratio
PLAT230_ALERT_2_C Hirshfeld Test Diff for    N1       --C36      .        6.0 s.u.
PLAT244_ALERT_4_C Low    'Solvent' Ueq as Compared to Neighbors of        C39 Check
PLAT244_ALERT_4_C Low    'Solvent' Ueq as Compared to Neighbors of        C41 Check
PLAT260_ALERT_2_C Large Average Ueq of Residue Including        N5      0.108 Check
PLAT340_ALERT_3_C Low Bond Precision on  C-C Bonds ..............      0.0054 Ang.
PLAT420_ALERT_2_C D-H Bond Without Acceptor  N1       --H1N      .     Please Check
PLAT420_ALERT_2_C D-H Bond Without Acceptor  N2       --H2N      .     Please Check
PLAT767_ALERT_4_C INS Embedded LIST 6 Instruction Should be LIST 4     Please Check
PLAT911_ALERT_3_C Missing FCF Refl Between Thmin & STh/L=    0.567         97 Report
```

Alert level G

```
PLAT154_ALERT_1_G The s.u.'s on the Cell Angles are Equal ..(Note)       0.03 Degree
PLAT910_ALERT_3_G Missing # of FCF Reflection(s) Below Theta(Min).          1 Note
PLAT933_ALERT_2_G Number of HKL-OMIT Records in Embedded .res File          8 Note
PLAT941_ALERT_3_G Average HKL Measurement Multiplicity ..........         2.8 Low
PLAT978_ALERT_2_G Number C-C Bonds with Positive Residual Density.          1 Info
```

0 **ALERT level A** = Most likely a serious problem - resolve or explain
1 **ALERT level B** = A potentially serious problem, consider carefully
15 **ALERT level C** = Check. Ensure it is not caused by an omission or oversight
5 **ALERT level G** = General information/check it is not something unexpected

2 ALERT type 1 CIF construction/syntax error, inconsistent or missing data
7 ALERT type 2 Indicator that the structure model may be wrong or deficient
9 ALERT type 3 Indicator that the structure quality may be low
3 ALERT type 4 Improvement, methodology, query or suggestion
0 ALERT type 5 Informative message, check

It is advisable to attempt to resolve as many as possible of the alerts in all categories. Often the minor alerts point to easily fixed oversights, errors and omissions in your CIF or refinement strategy, so attention to these fine details can be worthwhile. In order to resolve some of the more serious problems it may be necessary to carry out additional measurements or structure refinements. However, the purpose of your study may justify the reported deviations and the more serious of these should normally be commented upon in the discussion or experimental section of a paper or in the "special_details" fields of the CIF. checkCIF was carefully designed to identify outliers and unusual parameters, but every test has its limitations and alerts that are not important in a particular case may appear. Conversely, the absence of alerts does not guarantee there are no aspects of the results needing attention. It is up to the individual to critically assess their own results and, if necessary, seek expert advice.

Publication of your CIF in IUCr journals

A basic structural check has been run on your CIF. These basic checks will be run on all CIFs submitted for publication in IUCr journals (*Acta Crystallographica*, *Journal of Applied Crystallography*, *Journal of Synchrotron Radiation*); however, if you intend to submit to *Acta Crystallographica Section C* or *E* or *IUCrData*, you should make sure that full publication checks are run on the final version of your CIF prior to submission.

Publication of your CIF in other journals

Please refer to the *Notes for Authors* of the relevant journal for any special instructions relating to CIF submission.

PLATON version of 06/07/2023; check.def file version of 30/06/2023

Datablock DS_4_203c_0m_a - ellipsoid plot

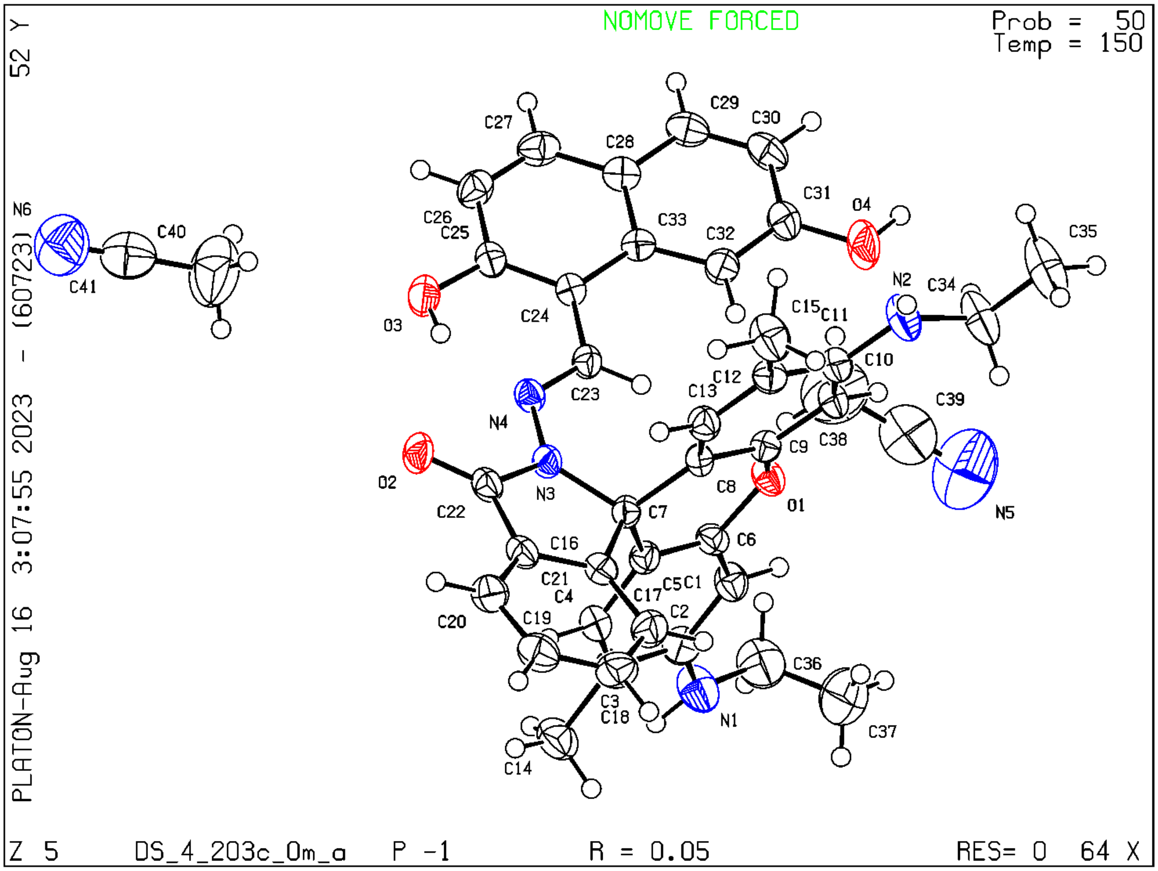

Supplement: Check Cif [file NIHMS1933183-supplement-Check_Cif.pdf]
